# Analysis of Listeria using exogenous volatile organic compound metabolites and their detection by static headspace–multi-capillary column–gas chromatography–ion mobility spectrometry (SHS–MCC–GC–IMS)

**DOI:** 10.1007/s00216-017-0375-x

**Published:** 2017-05-08

**Authors:** Carl Taylor, Fraser Lough, Stephen P. Stanforth, Edward C. Schwalbe, Ian A. Fowlis, John R. Dean

**Affiliations:** Department of Applied Sciences, Northumbria University, Newcastle upon Tyne, NE1 8ST UK

**Keywords:** Volatile organic compounds, Bacteria, *Listeria* spp., Static headspace–multi-capillary column–gas chromatography–ion mobility spectrometry (SHS–MCC–GC–IMS), GC–MS

## Abstract

*Listeria monocytogenes* is a Gram-positive bacterium and an opportunistic food-borne pathogen which poses significant risk to the immune-compromised and pregnant due to the increased likelihood of acquiring infection and potential transmission of infection to the unborn child. Conventional methods of analysis suffer from either long turn-around times or lack the ability to discriminate between *Listeria* spp. reliably. This paper investigates an alternative method of detecting *Listeria* spp. using two novel enzyme substrates that liberate exogenous volatile organic compounds in the presence of α-mannosidase and d-alanyl aminopeptidase. The discriminating capabilities of this approach for identifying *L. monocytogenes* from other species of *Listeria* are investigated. The liberated volatile organic compounds (VOCs) are detected using an automated analytical technique based on static headspace–multi-capillary column–gas chromatography–ion mobility spectrometry (SHS–MCC–GC–IMS). The results obtained by SHS–MCC–GC–IMS are compared with those obtained by the more conventional analytical technique of headspace–solid phase microextraction–gas chromatography–mass spectrometry (HS–SPME–GC–MS). The results found that it was possible to differentiate between *L. monocytogenes* and *L. ivanovii*, based on their VOC response from α-mannosidase activity.

## Introduction

*Listeria monocytogenes* is a pathogenic and teratogenic Gram-positive species of bacteria belonging to the genus Listeria, comprised of six distinct species as follows: *L. monocytogenes*, *L. innocua*, *L. ivanovii*, *L. seeligeri*, *L. welshimeri* and *L. grayi*. *L. monocytogenes* is the only species considered to be pathogenic to humans, although there are rare reports of cases of infection caused by *L. innocua* [1] and *L. ivanovii* [2]. *L. monocytogenes* poses a serious risk as a foodborne pathogen to the immunocompromised with most outbreaks coming from cooked meats, shellfish, prepared salads, ‘ready to eat’ foods and dairy products due to *L. monocytogenes*’ ability to survive conditions often adopted for the preservation of food, including a broad pH range, high salt concentrations and across a broad temperature range (0 to 45 °C) [3]. *L. monocytogenes* is often difficult to detect when present in low cell numbers [3], being able to persist in food products for extended periods of time; this is of concern in refrigerated products, where growth of *L. monocytogenes* can be favoured over other bacteria which may be present, leading to proliferation of *L. monocytogenes* in concentrations high enough to cause illness. Current detection methods for bacteria in foods often require prolonged sample incubation periods, with identification achieved by culture followed by, biochemical testing, or immunoassay which is often time-consuming [3]. Culture methods often lack the specificity to differentiate between pathogenic and non-pathogenic species of *Listeria* which pose no significant health risk; hence, a method for the rapid detection of *L. monocytogenes* that can differentiate species of *Listeria* as well as bacteria belonging to different genera is highly desirable. Recently, ion mobility spectrometry (IMS) has started to gain popularity in research within the pharmaceutical, microbiological and environmental fields with commercial instruments readily available [4]. Initially, the instrument, which has functionality comparable to that of a time-of-flight mass spectrometer (TOF MS), was used mainly by the military and in airports for the rapid detection of chemical warfare agents, explosives and narcotics [5], a task that was well suited to it due to its high sensitivity, portability and rapid result turnover. However, it is these characteristics that also make it a suitable candidate for the rapid identification of *L. monocytogenes*.

IMS in recent years has been applied to the rapid analysis of bacteria and other microorganisms using numerous different approaches. One such approach is to analyse the headspace of bacteria directly to detect microbial volatile organic compounds (MVOCs) released by the bacteria. This approach was adopted [6] for the detection of mould, by use of a hand-held IMS to analyse VOCs released by mould in rooms. The authors recorded the concentration of investigated MVOCs up to concentrations of 9 ppm_V_ with estimated limits of detection (LOD) ranging from 1 to 52 ppb_V_. The approach was further demonstrated by Kunze et al. [7] who used multicapillary column (MCC) coupled to IMS to detect pathogenic bacteria (*Pseudomonas aeruginosa* and *Escherichia coli*). They identified a range of compounds including aldehydes, alcohols and ketones, using an in-house database, commonly associated with bacteria breakdown. Similarly, other researchers (Vinopal et al. [8]) have used IMS as a fingerprinting approach, in both the positive and negative ionisation modes, to obtain combined plasmagrams in order to circumvent the problem of the similarity of produced MVOCs. The investigation which included over 200 strains and species of bacteria found that reproducible and original plasmagrams were obtainable in each case [8]. This approach was also adopted by Jünger et al. [9] who explored the specificity of naturally released VOCs from 15 different strains of bacteria in both positive and negative modes; they highlighted that some VOCs were unique to the bacteria investigated. The importance of obtaining data in both positive and negative modes was emphasised.

An alternative approach to determine VOCs exploits the presence of extracellular enzymes within bacteria. Enzymes present can cleave added substrates to liberate either VOCs, colorimetric or fluorescent compounds which can subsequently be detected with the naked eye or via an analytical instrument (e.g. fluorimeter or GC–MS). This approach has been demonstrated by Dean and co-workers in two separate papers [3, 10] applying this approach for the detection of *L. monocytogenes* in milk and other pathogenic bacteria. In the former paper [3], 2-nitrophenol and 3-fluoroaniline were chosen as the target VOCs due to their unlikely natural occurrence in biological systems as well as their ability to be detected by solid phase microextraction (SPME) GC–MS. The use of 2-nitrophenyl-β-d-glucopyranoside and 2-[(3-fluorophenyl)-carbamoylamino] acetic acid substrates targets the β-glucosidase and hippuricase enzymes, respectively. The results obtained demonstrated the effectiveness of the approach to detect *L. monocytogenes* in milk samples. In the latter study [10], modified agarose gel was used to trap exogenous VOCs produced by pathogenic bacteria including *Escherichia coli*, *Klebsiella pneumoniae*, *Pseudomonas fluorescens*, *Enterococcus faecium* and *Streptococcus agalactiae*. Identification was performed visually by means of chemical transformation of VOCs to coloured compounds within the prepared gel. The added advantage of exploiting extracellular enzymes of bacteria is that it increases the selectivity of the technique towards the targeted bacteria, as substrates can be selected which are not hydrolysed by bacteria that share a similar MVOC profile to that of the bacteria of interest.

The aim of this paper is to investigate the potential application of static headspace–multi-capillary column–gas chromatography–ion mobility spectrometry (SHS–MCC–GC–IMS) to the identification of *Listeria* species. Specifically, this paper aims to (a) confirm the presence of *Listeria* species via their inherent α-mannosidase activity, (b) differentiate *Listeria monocytogenes* from other Listeria’s and (c) differentiate the pathogenic bacteria *Listeria monocytogenes* from *Listeria ivanovii*. The basis of the approach is to generate exogenous volatile organic compounds by the hydrolytic enzyme activities of *L. monocytogenes*, or non-pathogenic *Listeria* spp. upon two enzyme substrates (i.e. benzyl-α-d-mannopyranoside and d-alanyl-3-fluoroanilide) which will liberate either benzyl alcohol or 3-fluoroaniline in the presence of α-mannosidase and d-alanine aminopeptidase activities, respectively. It has been reported that *L. monocytogenes* does not normally have d-alanyl aminopeptidase activity [11] but does possess α-mannosidase activity [12]. In contrast, d-alanine aminopeptidase activity is produced by non-pathogenic *Listeria* species, which can also variably produce the α-mannosidase enzyme. The results of the exogenous VOC analysis will be compared via headspace sampling using solid phase microextraction (SPME) coupled to a gas chromatography mass spectrometer (GC–MS).

## Materials and methods

### Reagents/chemicals

The following analytical grade reagents were obtained from commercial suppliers. Tetrahydrofuran, dichloromethane, sodium bicarbonate, magnesium sulphate, methanol, ethyl acetate and hydrochloric acid were obtained from Fisher Scientific (Loughborough, UK). Acetone was acquired from Sigma-Aldrich (Dorset, UK). 3-Fluoroaniline was obtained from Fluorochem (Derbyshire, UK). Benzyl alcohol was obtained from Acros Organics (Geel, Belgium). 1-Methyl-2-pyrrolidinone, isobutyl chloroformate, n-(tert-butoxycarbonyl)-d-alanine and citric acid were obtained from Alfa Aesar (Lancashire, UK). n-Methylmorpholine was obtained from Lancaster Synthesis (Lancaster, UK). Benzyl-α-d-mannopyranoside was obtained from Santa Cruz Biotechnology (Heidelberg, Germany). Listeria enrichment broth base CM0862 was obtained from Oxoid Limited (Basingstoke, UK).

### Instrumentation

A static headspace–multi-capillary column–gas chromatography–ion mobility spectrometer (SHS–MCC–GC–IMS) manufactured by G.A.S.—Gesellschaft für Analytische Sensorsysteme mbH (Dortmund, Germany) was used [13]. The SHS–MCC–GC–IMS was fitted with an automatic sampler unit (CTC-PAL; CTC Analytics AG, Zwin-gen, Switzerland) and a heated gas-tight syringe. A multi-capillary column (MCC) (Multichrom, Novosibirsk, Russia) was used for the chromatographic separation. The MCC comprised a stainless steel tube, 20 cm × 3 mm ID, containing approximately 1000 parallel capillary tubes, 40 μm ID, coated with 0.2-μm film thickness of stationary phase (Carbawax 20 M). Atmospheric pressure ionisation is generated by a tritium (^3^H) solid-state bonded source (β-radiation, 100–300 MBq with a half-life of 12.5 years). The IMS has a drift tube length of 50 mm. Separation in the IMS drift tube is achieved by applying an electric field of 2 kV to the ionised volatiles in a pulsed mode using an electronic shutter opening time of 100 μs. The drift gas was N_2_ (99.998%) with a drift pressure of 101 kPa (ambient pressure). Samples were run under the following operating conditions: incubation conditions (time, 5 min and temperature, 40 °C), MCC–IMS conditions (syringe temperature, 85 °C; injection temperature, 80 °C; injection volume, 0.5 mL; column temperature, 50 °C; and column carrier gas flow programme rate, 15 mL/min to 150 mL/min (in step-wise increments of 2 mL/min every 6 s; the total flow programme is complete in 6.9 mins with 69 steps)); and IMS conditions (temperature, 50 °C and drift gas flow rate, 500 mL/min). The total analysis time was 21 min. All data was acquired in the positive ion mode and each spectrum is formed with the average of 42 scans. All data are processed using the LAV software (version 2.0.0, G.A.S). The software package enables both two- and three- dimensional data visualisation plots. Following injection of the SPME fibre, 1 mL of the previously equilibrated sample was transferred by pipette into a 20-mL sterile headspace vial (Sage Analytical Ltd., Heywood, UK) and capped with a sterile bimetallic crimp cap (Sage Analytical Ltd., Heywood, UK) prior to sampling and analysis by MCC–GC–IMS. The experimental procedure has previously been reported for analysis of VOCs associated with malodour in laundry [13].

Gas chromatography/mass spectrometry (GC/MS) analysis was performed on a Thermo Finnegan Trace GC Ultra and Polaris Q ion trap mass spectrometer (Thermo Scientific, UK) with the Xcalibur 1.4 SR1 software. Separation of VOCs was carried out using a 30 m × 0.25 mm × 0.25 μm VF-WAXms polar GC column (Agilent Technologies, Wokingham, UK). Separation of bacterial VOCs was achieved using the following temperature program: initial 50 °C with a 3-min hold, ramped to 250 °C at 12.5 °C/min and then held for 2 min. The split-splitless injection port was held at 230 °C for desorption of volatiles in split mode at a split ratio of 10:1. Helium was used as the carrier gas at a constant flow rate of 1.0 mL/min. MS parameters were as follows: full-scan mode with scan range of 33–500 amu at a rate of 0.50 scan/s. The ion source temperature was 260 °C with an ionising energy of 70 eV and a mass transfer line of 250 °C. Identification of VOCs was achieved using the National Institute of Standards and Technology (NIST) reference library (NIST Mass spectral library, version 2.0a, 2001) as well as the comparison of the retention times and mass spectra of authentic standards.

A 85-μm polyacrylate (PA) SPME fibre (Sigma-Aldrich, Poole, UK) was used for extraction of bacterial volatiles; the fibre was conditioned prior to use at 230 °C for 60 min in the GC injection port, followed by a GC oven temperature ramp to 250 °C for 15 min to remove SPME fibre-related impurities from the column. Fibres were used with a manual holder and not used beyond the manufacturer’s recommended number of injections. Samples were taken individually from the incubator set at 37 °C and placed in a water bath at 37 °C for 15 min to ensure full temperature equilibration. VOCs were collected by SPME fibre for 10 min (no stirring) and thermal desorption for 3 min at 230 °C in the injection port.

Data analysis using principal component analysis was done using R R: a language and environment for statistical computing (R Foundation for Statistical Computing, Vienna, Austria. URL https://www.R-project.org/).

### Synthesis of substrate d-alanyl-3-fluoroanilide

**Scheme 1:**
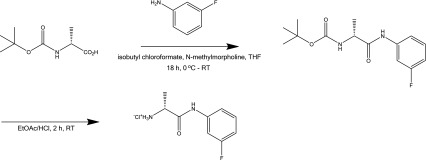
Synthesis of d-alanyl-3-fluoroanilide

3-Fluoroaniline (2.63 mmol) was dissolved in 20 mL of dry THF and cooled to −5 °C in an ice/salt bath. n-(Tert-butoxycarbonyl)-d-alanine (2.63 mmol) was dissolved separately with n-methylmorpholine (2.63 mmol) in 20 mL of dry THF, and the solution cooled to −5 °C in an ice/salt bath. Isobutyl chloroformate (2.63 mmol) was added to the n-boc-d-Ala-OH solution and stirred for 2 min, followed by slow addition of the 3-fluoroaniline solution. The mixture was stirred and allowed to reach room temperature overnight. The THF was removed under reduced pressure and the resulting solid was dissolved in 20 mL DCM and washed with saturated sodium bicarbonate solution, 0.1-M citric acid solution and brine. The organic layer was dried over MgSO_4_ and the solvent removed under reduced pressure. The crude solid was purified by dissolution in a minimal amount of methanol, followed by dropwise addition of water until the solution appeared cloudy, white crystals formed upon cooling in an ice bath which were obtained by vacuum filtration. De-protection was achieved by stirring in 5 mL of ethyl acetate saturated with hydrogen chloride gas for 2 h. The title compound precipitated out of solution and was obtained as a white powder by vacuum filtration (Scheme 1).

### Analytical data

n-Boc-d-alanyl-3-fluoroanilide: yield 0.5173 g (69.7%); mp 163 °C; IR (ATR) cm^−1^: 3344 (m, N–H), 2984 (w, C–H), 1675 (s, C=O); ^1^H NMR (d_6_-DMSO, 400 MHz) δ: 10.1 (s, 1H, NH), 7.56 (dd, J = 12, 2 Hz, 1H, ArH), 7.30 (m, 2H, ArH, NH), 7.11 (d, J = 6.8 Hz, 1H, ArH), 6.83 (m, 1H, ArH), 4.05 (quin, J=, 1H, CH), 1.34 (s, 9H, 3 × CH_3_), 1.21 (d, J = 7.2 Hz, CH_3_); ^13^C NMR (d_6_-DMSO, 100 MHz) δ: 172, 164 (d, J = 239.4 Hz, C–F), 156, 141, 131, 115, 110 (d, J = 21.0 Hz, ArC), 106 (d, J = 25.7 Hz, ArC), 79, 51, 29, 18; LRMS (ESI) for C_14_H_19_FN_2_O_3_ calculated (M + H) *m*/*z* 283.1458, found *m*/*z* 283.1457.

d-Alanyl-3-fluoroanilide: yield 0.3176 g (79.3%); mp 208 °C; IR (ATR) cm^−1^: 3087 (m, ArC–H), 2855 (m, C–H), 1673 (s, C=O); ^1^H NMR (d_6_-DMSO, 400 MHz) δ: 11.21 (s, 1H, NH), 8.39 (s, 3H, NH3), 7.60 (dd, J = 11.6, 1.2 Hz, 1H, ArH), 7.40 (m, 2H, 2 × ArH), 6.90 (t, J = 7.8 Hz, 1H, ArH), 4.07 (s, 1H, C–H), 1.44 (d, J = 6.8 Hz, 3H, CH3); ^13^C NMR (d_6_-DMSO, 100 MHz) δ: 169, 163 (d, J = 241.2 Hz, ArC), 131 (d, J = 9.5 Hz, ArC) 116, 111 (d, J = 21.0 Hz, ArC), 107 (d, J = 26.7 Hz, ArC), 49, 18; HRMS (NSI) for C_9_H_12_FN_2_O calculated (M+) *m*/*z* 183.0934, found *m*/*z* 183.0928.

### Preparation of *Listeria* samples

Listeria enrichment broth base (3.6 g) was dissolved in 100 mL of deionised water and 10.0 mL transferred to 7 × 20-mL screw-cap vials and autoclaved along with 20-mL headspace vials and crimp cap. Meanwhile, 5.0 mg of each substrate was weighed into a 1.5-mL sterilised microcentrifuge tube and dissolved in 250 μL of deionised water to give 20,000-ppm standards. Then, 50 μL of each 20,000-ppm standard was added to three of the vials. A 0.5 McFarland standard was then prepared by transferring the desired culture (sub-cultured onto tryptone soya agar 24 h prior to preparation) to a screw-lock vial containing 10.0 mL of sterile broth and measuring the absorbance measured at 600 nm to determine turbidity until the value obtained from the broth blanked spectrometer read 0.132. Finally, 100 μL of this standard was added to six of the vials to yield approximately 1 × 10^6^ CFU mL^−1^ of each bacteria i.e. *L. monocytogenes* (NCTC 11994), *L. monocytogenes* (NCTC 10357), *L. grayi* (NCTC 10815), *L. seeligeri* (NCTC 11256), *L. welshimeri* (NCTC 11857), *L. ivanovii* (NCTC 11846) and *L. innocua* (NCTC 11288). For the analyses, one blank, three pure cultures and three cultures with substrates were then incubated at 37 °C for 24 h prior to analysis.

### Identification of isolated bacteria

NCTC strains of *Listeria* spp. were sub-cultured onto non-selective tryptone soya agar (TSA). The plates were then incubated at 37 °C for 24 h. The fresh plates were examined, and a single colony of each was picked with a sterile toothpick and deposited onto a polished stainless steel matrix-assisted laser desorption ionisation (MALDI) target plate. The matrix solution was prepared by dissolving 10 mg of α-cyano-4-hydroxycinnamic acid in 475 μL distilled water/500 μL acetonitrile/25 μL trifluoroacetic acid; the colony material was overlaid with 1.0 μL of matrix solution and allowed to air-dry. Matrix-assisted laser desorption ionisation–time-of-flight mass spectrometry (MALDI–TOF MS) analyses were conducted using a Bruker Biotyper (Bruker, Coventry, UK) over a mass range of 2000–20,000 Da. The Bruker taxonomy library was used to confirm identification of the bacteria.

## Results and discussion

**Fig. 1:**
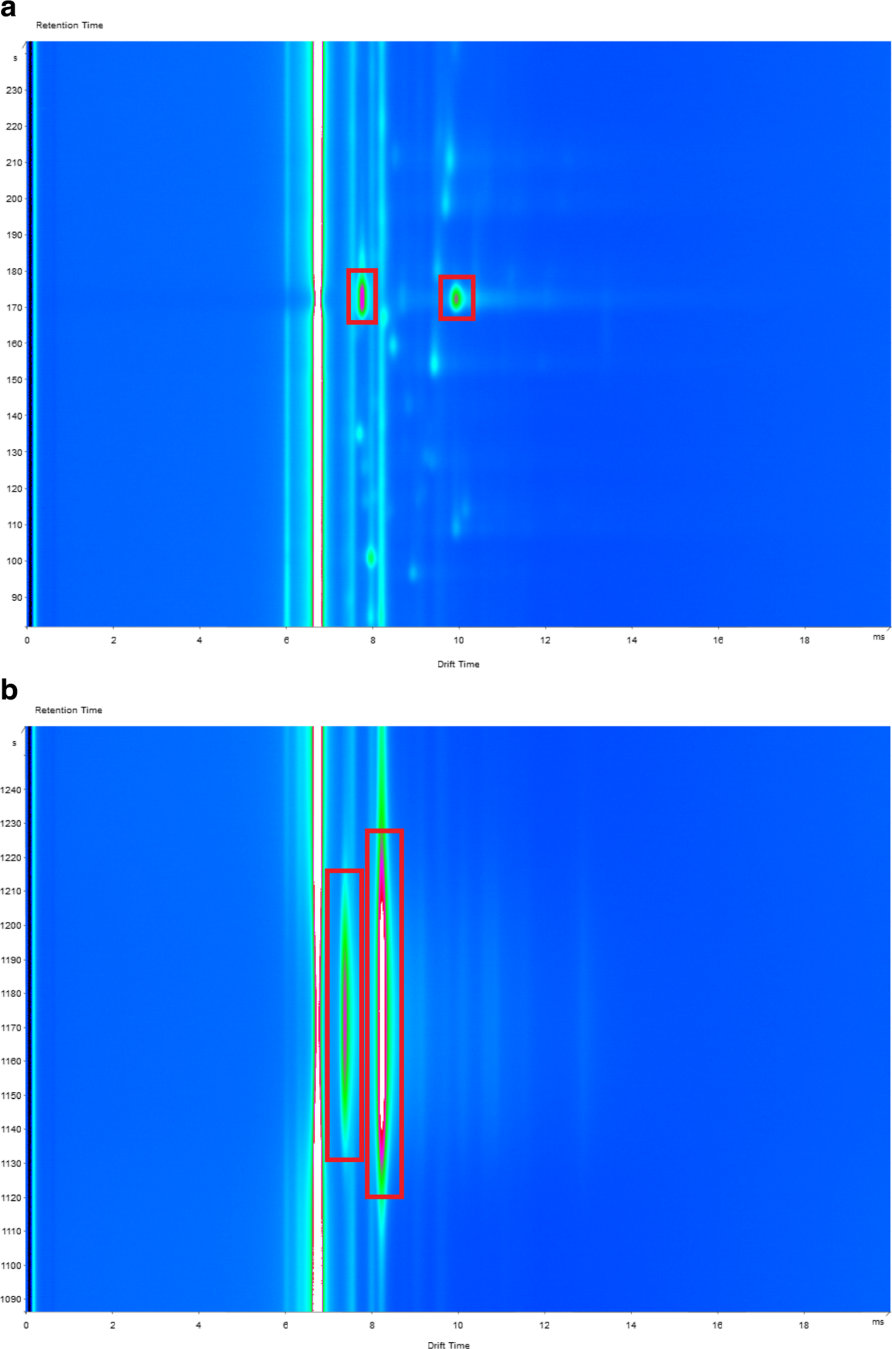
SHS–MCC–GC–IMS of VOCs benzyl alcohol (**a**) and 3-fluoroaniline (**b**)

Initially, the analytical performance of the two enzyme substrate exogenous VOCs, by SHS–MCC–GC–IMS, was done (Table 1). Benzyl alcohol had both a monomer and a dimer with a retention time of 173.4 ± 0.4 s and drift times of 7.79 ± 0.01 and 9.97 ± 0.01 ms, respectively (Fig. 1a), and 3-fluoroaniline had both a monomer and dimer with a retention time of 1179 ± 0.9 s and drift times of 7.41 ± 0.01 and 8.23 ± 0.01 ms, respectively (Fig. 1a). The relative drift time (*t*_r_drift) for each of the two VOCs was calculated using the following Eq. (1) [13].

**Table 1:** Peak identification data for VOCs

Compound name	Compound clusters	Retention time (s) mean ± SD (*n* = 3)	Drift time (ms) mean ± SD (*n* = 3)		Relative drift time (ms) mean ± SD (*n* = 3)		Normalised reduced ion mobility K_0_ (cm^2^ V^−1^ s^−1^) mean ± SD (*n* = 3)	
			Monomer	Dimer	Monomer	Dimer	Monomer	Dimer
Benzyl alcohol	Monomer + dimer	173.4 ± 0.4	7.79 ± 0.01	9.97 ± 0.01	1.15 ± 0.00	1.47 ± 0.00	1.36 ± 0.00	1.09 ± 0.00
3-Fluoroaniline	Monomer + dimer	1179 ± 0.9	7.41 ± 0.01	8.23 ± 0.01	1.09 ± 0.00	1.21 ± 0.00	1.43 ± 0.00	1.28 ± 0.00
Reactant ion peak (RIP)			6.78 ± 0.02^a^				1.56 ± 0.02^a^	

^a^ *n* = 20

1 $$ {t}_{\mathsf{r}}\mathsf{drift}\kern0.5em =\kern0.5em {t}_{\mathsf{d}}\kern0.5em /\kern0.5em {t}_{\mathsf{d}}\mathsf{RIP}, $$

where *t*_d_ is the measured drift time of the VOC and *t*_d_RIP is the drift time of the reactant ion peak (RIP), and reported in Table 1. The use of the reactant ion peak as an internal reference point is analogous to the use of the retention time of an unretained component in gas (or high-performance liquid) chromatography to calculate the capacity factor. Additionally, the normalised reduced ion mobility (Ko, cm^2^ V^−1^ s^−1^) can be calculated for each VOC. In order to do this, the normalised reduced ion mobility for the RIP (Ko(RIP)) must first be calculated (using Eq. 2):

2 $$ \mathrm{Ko}\left(\mathrm{RIP}\right)=\kern0.75em \left[\right(\frac{L2}{} E\times t\mathrm{D}\left(\mathrm{RIP}\left)\right)\times \left( P\frac{}{} P\mathrm{o}\right)\times \left( T\mathrm{o}\frac{}{} T\right)\right]\frac{L^2}{E{ t}_{\mathrm{D}}}, $$

where *L* is the length of the drift region (cm), *E* is the electrical field strength (V), *t*_D_(RIP) is the drift time (s) of the RIP, *P* is the pressure of the drift gas (hPa), *P*_o_ is the standard atmospheric pressure (1013.2 hPa), *T* is the temperature of the drift gas (K) and *T*_o_ is the standard temperature (273 K). The normalised reduced ion mobility for the RIP (Ko(RIP) was experimentally determined to be 1.56 ± 0.02 cm^2^ V^−1^ s^−1^ (*n* = 20) (Table 1).

Once the Ko(RIP) values has been experimentally determined, the normalised reduced ion mobility (Ko) for the VCs, in units of cm^2^ V^−1^ s^−1^, can be calculated as follows (Eq. 3):

3 $$ {\mathsf{K}}_{\mathsf{0}}{\left(\mathsf{VOC}\right)}_{=}{F}_{\mathsf{IMS}}/{t}_{\mathsf{D}}\left(\mathsf{VOC}\right), $$

where *F*_IMS_ is the IMS factor (cm^2^ V^−1^) derived as follows: *F*_IMS_ = K_0_(RIP) × *t*_D_(RIP), and *t*_D_(VOC) is the drift time (ms) of the VOC. The derived normalised reduced ion mobilities for the two VOCs as their monomer and dimer are shown in Table 1.

**Fig. 2:**
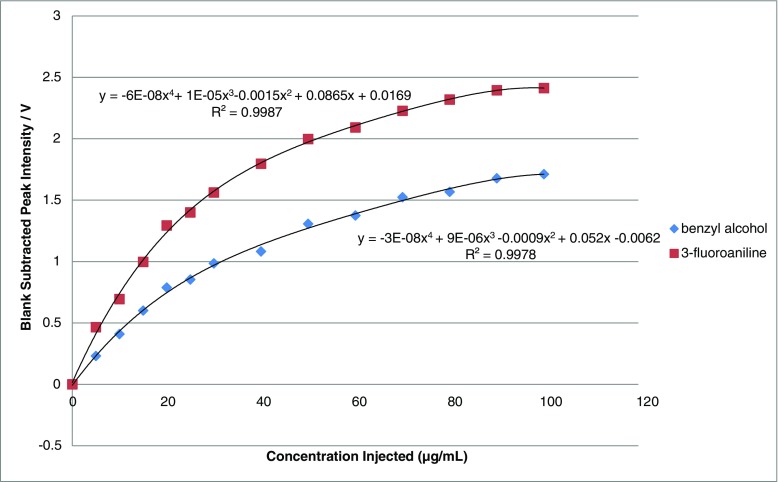
Calibration graph for benzyl alcohol and 3-fluoroaniline

Subsequently, the calibration data for each of the two exogenous VOCs was determined (Fig. 2). Non-linearity was determined for benzyl alcohol and 3-fluoroaniline over the concentration range 0–100 μg/mL. However, linear calibration graphs, for both VOCs, were obtained over the concentration range of 0–20 μg/mL (Table 2), with typical correlation coefficients, *R* ^2^, of >0.99, irrespective of VOC. This data was compared to the analysis of the VOCS using SPME–GC–MS which gave linear calibration graphs over the concentration range of 0–100 μg/mL, and with typical correlation coefficients, *R* ^2^, of >0.99, irrespective of VOC. The limit of detection (LOD) and limit of quantitation (LOQ), based on 3 or 10 × standard deviation of the blank, respectively, were determined. It can be seen that the pre-concentration step associated with the headspace sampling by SPME allows either ×4 or ×10 lower LOD to be obtained for benzyl alcohol or 3-fluoroaniline, respectively (Table 2).

**Table 2:** Calibration data for VOCs by SHS–MCC–GC–IMS and SPME–GC–MS

Compound name	Analytical technique	Non-linear				Linear					
		Range μg/mL	*N*	Equation	*R* ^2^	Range μg/mL	*N*	Equation	*R* ^2^	LOD μg/mL	LOQ μg/mL
Benzyl alcohol	SHS–MCC–GC–IMS	0–100	14	*y* = −3E-08*x* ^4^ + 9E-06*x* ^3^ – 0.0009*x* ^2^ + 0.052*x* − 0.0062	0.9978	0–20	5	*y* = 0.039*x* − 0.0166	0.9981	2.4	9.5
	SPME–GC–MS	NA				0–100	11	*y* = 13,260*x* − 13,762	0.9894	0.6	1.7
3-Fluoroaniline	SHS–MCC–GC–IMS	0–100	14	*y* = −6E-08*x* ^4^ + 1E − 05*x* ^3^ – 0.0015*x* ^2^ + 0.0865*x* + 0.0169	0.9987	0–20	5	*y* = 0.0624*x* + 0.066	0.9875	0.5	2.2
	SPME–GC–MS	NA				0–100	11	*y* = 90,862*x* + 11,901	0.9974	0.05	0.15

Analytical data is based on Σ monomer + dimer

*NA* not applicable

### *Listeria* analysis

**Fig. 3:**
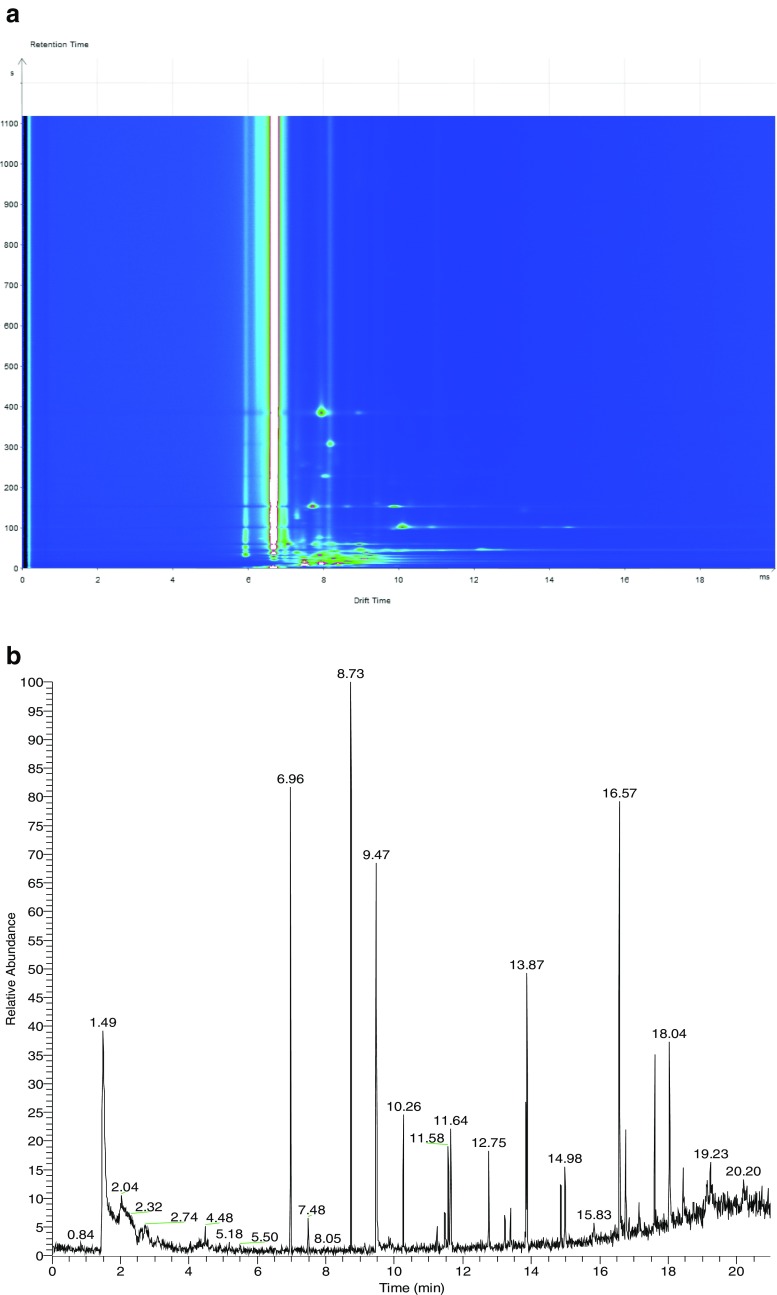
Analysis of a pure culture of *Listeria monocytogenes 10357* by SHS–MCC–GC–IMS (**a**) and HS–SPME–GC–MS (**b**)

Analysis by both chromatographic techniques i.e. SHS–MCC–GC–IMS and HS–SPME–GC–MS yielded several more peaks produced by the pure cultures of all *Listeria* species. Example chromatograms of the pure cultures of *Listeria monocytogenes* (NCTC 10357) are shown in Fig. 3. Figure 3a shows the typical chromatogram obtained by SHS–MCC–GC–IMS highlighting the relative ‘cleanness’ of the chromatogram at retention times >200 s (For reference, the retention times of benzyl alcohol and 3-fluoroaniline by SHS–MCC–GC–IMS are 173 and 1179 min, respectively). While Fig. 3b shows a typical chromatogram obtained by HS–SPMS–GC–MS. In this situation, the relative ‘complexity’ of the chromatogram is highlighted with potential interferences from volatiles present in the growth media (broth), as well as SPME fibre and GC column components (For reference, the retention times of benzyl alcohol and 3-fluoroaniline in HS–SPME–GC–MS are 13.54 and 13.69 min, respectively). These peaks were shared in-common by all the species but with varying degrees of intensities. However, it was concluded that this approach was not useful; by SHS–MCC–GC–IMS, peak identification is not currently possible while by HS–SPME–GC–MS, peak identification would need to be authenticated using a known standard (and mass spectral corroboration).

**Scheme 2:**
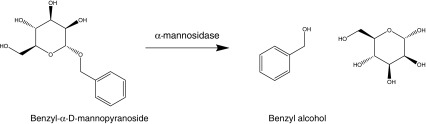
Benzyl alcohol formation from substrate

**Scheme 3:**
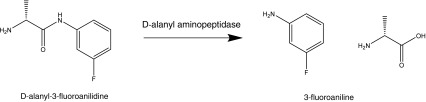
3-Fluoroaniline formation from substrate

The analytical results for the determination of benzyl alcohol and 3-fluoroaniline after addition of the specific enzyme substrates to pure cultures of *Listeria*, and incubation, are shown in Table 3. The results represent the mean and individual values from three separate analyses of the bacteria. It is noted in general terms that the concentration of benzyl alcohol detected by SHS–SPME–GC–MS is often greater than that determined by SHS–MCC–GC–IMS; this is not unexpected given the pre-concentration step incorporated within this approach i.e. SPME. This effect is most noticeable at the higher detected concentrations. In general terms, it is evident that, in all cases, either analytical technique has determined a concentration of benzyl alcohol after addition of the enzyme substrate benzyl-α-d-mannopyranoside (Scheme 2). The determination of benzyl alcohol is indicative of α-mannosidase. In contrast, and almost exclusively, both analytical techniques have not detected 3-fluoroaniline, after addition of the enzyme substrate d-alanyl-3-fluoroanilide (Scheme 3), thereby indicating the absence of d-alanyl aminopeptidase activity. As might be expected with the data obtained for benzyl alcohol (Table 3), the within-sample variation (*n* = 3) for each *Listeria spp.* is fair: a mean 28.2%RSD, with a range of 7.1–89.1%RSD for SHS–MCC–GC–IMS and a mean 24.0%RSD, with a range of <1.0–86.3%RSD for HS–SPME–GC–MS. Whereas the inter-*Listeria spp*. variation is more variable, as might be expected: a mean of 32.1%RSD, with a range of 2.3–72.9%RSD (*n* = 3) for SHS–MCC–GC–IMS and a mean of 37.3%RSD, with a range of 4.0–150%RSD (*n* = 3) for HS–SPME–GC–MS at the 1 × 10^6^ CFU/mL.

**Table 3:** Listeria analysis: VOC data

Listeria species	SHS–MCC–GC–IMS						SPME–GC–MS					
	Benzyl alcohol (μg/mL)			3-Fluoroaniline (μg/mL)			Benzyl alcohol (μg/mL)			3-Fluoroaniline (μg/mL)		
	Analysis 1	Analysis 2	Analysis 3	Analysis 1	Analysis 2	Analysis 3	Analysis 1	Analysis 2	Analysis 3	Analysis 1	Analysis 2	Analysis 3
*L. monocytogenes (NCTC 11994)*	9.4 (0.1; 11.7; 16.3)	12.9 (11.8; 13.9; 12.8)	9.7 (8.6; 10.8; 9.8)	ND (ND; ND; ND)	ND (ND; ND; ND)	ND (ND; ND; ND)	31.0 (30.0; 29.8; 33.2)	30.8 (29.7; 29.6; 32.9)	26.0 (30.6; 21.5)	ND (ND; ND; ND)	ND (ND; ND; ND)	ND (ND; ND; ND)
*L. monocytogenes (NCTC 10357)*	6.5 (7.1; 6.9; 5.6)	6.7 (7.3; 8.9; 4.0)	6.4 (5.9; 6.8; 6.3)	ND (ND; ND; ND)	ND (ND; ND; ND)	ND (ND; ND; ND)	25.7 (28.0; 18.6; 30.5)	20.7 (19.0; 21.7; 21.5)	19.5 (30.1; 13.0; 15.3)	ND (ND; ND; ND)	0.2 (0.2; 0.2; 0.2)	ND (ND; ND; ND)
*L. grayi (NCTC 10815)*	5.5 (5.8; 5.7; 4.9)	6.2 (4.9; 6.9; 6.8)	10.1 (11.9; 9.6; 8.7)	ND (ND; ND; ND)	ND (ND; ND; ND)	ND (ND; ND; ND)	31.1 (31.5; 27.3; 34.4)	28.3 (28.1; 27.7; 29.1)	30.4 (30.2; 23.6; 37.3)	ND (ND; ND; ND)	ND (ND; ND; ND)	0.1 (0.1; ND; 0.1)
*L. seeligeri (NCTC 11256)*	1.8 (2.6; 2.5; 0.3)	1.9 (1.3; 2.5; 1.9)	1.7 (1.7; −; 1.6)	ND (ND; ND; ND)	ND (ND; ND; ND)	ND (ND; ND; ND)	0.7 (1.0; 0.6; 0.5)	0.4 (0.7; 0.4; 0.2)	1.0 (0.8; 1.0; 1.1)	ND (ND; ND; ND)	ND (ND; ND; ND)	0.1 (0.2; 0.1; 0.1)
*L. welshimeri (NCTC 11857)*	20.0 (20.6; 17.5; 21.8)	22.9 (22.6; 19.4; 26.7)	2.5 (3.8; 1.3; 2.4)	ND (ND; ND; ND)	ND (ND; ND; ND)	ND (ND; ND; ND)	34.8 (30.7; 35.6; 38.1)	29.9 (27.5; 36.0; 26.1)	36.4 (36.5; 37.0; 35.9)	ND (ND; ND; ND)	ND (ND; ND; ND)	ND (ND; ND; 0.1)
*L. ivanovii (NCTC 11846)*	1.3 (0.7; 1.9; 1.2)	3.4 (2.7; 2.8; 4.8)	2.0 (1.1; 2.9; −)	ND (ND; ND; ND)	ND (ND; ND; ND)	ND (ND; ND; ND)	0.7 (0.5; 0.7; 0.9)	0.5 (0.5; 0.5; 0.5)	0.9 (0.8; 0.9; 0.9)	ND (ND; ND; ND)	ND (ND; ND; ND)	0.1 (0.1; 0.1; 0.1)
*L. innocua (NCTC 11288)*	5.3 (6.9; 5.4; 3.6)	4.3 (4.6; 3.3; 5.1)	2.0 (2.2; 1.8; 2.2)	ND (ND; ND; ND)	ND (ND; ND; ND)	ND (ND; ND; ND)	1.5 (1.0; 2.9; 0.5)	0.6 (0.5; 0.9; 0.5)	21.5 (9.7; 23.6; 31.3)	ND (0.1; ND; ND)	ND (ND; ND; 0.1)	0.1 (0.1; 0.1; 0.2)

Mean (individual replicate analysis)

**Fig. 4:**
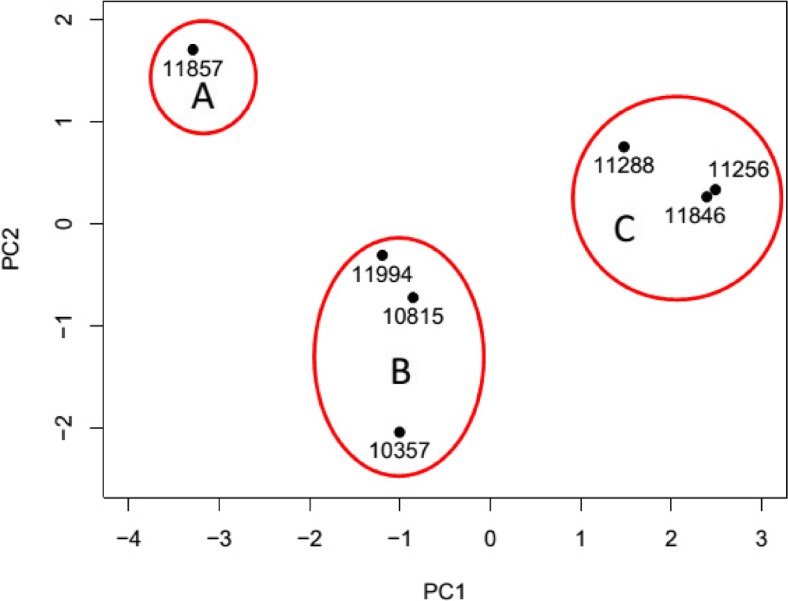
Principal component analysis of VOC data, irrespective of analytical technique

To further interrogate the data, a statistical method was required to emphasise variation and to visualise any patterns within the dataset; on that basis, principal component analysis (PCA) was selected. The PCA data (Fig. 4) was obtained using the whole dataset as shown in Table 3. Figure 4 shows the results of the PCA with respect to principal component (PC) 1 and PC2. PC1 identified 58.5% while PC2 17.6% of the data variance. It is evident that two distinct regions can be identified within the PCA profile (Fig. 4). Region A included the following bacteria: *L. welshimeri* (NCTC 11857); region B: *L. monocytogenes* (NCTC 11994), *L. monocytogenes* (NCTC 10357) and *L. grayi* (NCTC 10815); while region C: *L. seeligeri* (NCTC 11256), *L. ivanovii* (NCTC 11846) and *L. innocua* (NCTC 11288). However, it was concluded that strong similarities, in terms of their VOC concentrations, exist between the bacteria in regions A and B, and that a distinct difference is noted with respect to region C.

It has been previously reported [14] that α-mannosidase activity can be used to differentiate the pathogenic bacteria *L. monocytogenes* (a positive response) and *L. ivanovii* (a negative response). These results (Fig. 4) concur with this finding with the added advantage of speed and sensitivity of detection of the liberated VOC i.e. benzyl alcohol. Post-culturing the detection of benzyl alcohol was completed in 21 min. Interestingly, it is expected that *Listeria* spp. apart from *L. monocytogenes* are expected to illustrate a positive response for d-alanyl aminopeptidase activity [11, 14], in this situation, liberating the exogenous VOC 3-fluoroaniline. Unfortunately, this is not the case apart from an occasional sporadic detection of trace concentrations of 3-fluoroaniline by the more sensitive analytical technique i.e. HS–SPME–GC–MS (Table 3). This may be due to the duration of the microbiological incubation step not allowing sufficient time for the enzyme to be activated. So while the microbiological incubation time period was fixed at 24 h in this research, some bacteria require up to 72 h to produce a positive response using culturing methods. Also, the measured d-alanyl aminopeptidase activity may be low due to differing assay conditions and enzyme substrate to those reported in the literature [11]. It is noted that other workers have sought to differentiate *L. monocytogenes* from other Listeria’s based on their esterase activity [15].

Further work would seek to apply the developed methodology for the detection of *Listeria spp.* in food matrices e.g. milk samples. The inclusion of the enzyme substrates within a liquid matrix, followed by overnight incubation, would allow the potential to determine *Listeria spp.* based on the generation of exogenous VOCs. The presence of competing bacteria in real samples could be potentially controlled by the inclusion of antibiotics.
